# An Optical Fiber Viscometer Based on Long-Period Fiber Grating Technology and Capillary Tube Mechanism

**DOI:** 10.3390/s101211174

**Published:** 2010-12-08

**Authors:** Jian-Neng Wang, Jaw-Luen Tang

**Affiliations:** 1 Department of Construction Engineering, National Yunlin University of Science and Technology, Douliou 64002, Taiwan; 2 Department of Physics, National Chung Cheng University, Chia-Yi 62102, Taiwan

**Keywords:** long-period fiber grating (LPFG), sensor, viscosity, asphalt, wavelength shift, refractive index (RI)

## Abstract

This work addresses the development and assessment of a fiber optical viscometer using a simple and low-cost long-period fiber grating (LPFG) level sensor and a capillary tube mechanism. Previous studies of optical viscosity sensors were conducted by using different optical sensing methods. The proposed optical viscometer consists of an LPFG sensor, a temperature-controlled chamber, and a cone-shaped reservoir where gravitational force could cause fluid to flow through the capillary tube. We focused on the use of LPFGs as level sensors and the wavelength shifts were not used to quantify the viscosity values of asphalt binders. When the LPFG sensor was immersed in the constant volume (100 mL) AC-20 asphalt binder, a wavelength shift was observed and acquired using LabVIEW software and GPIB controller. The time spent between empty and 100 mL was calculated to determine the discharge time. We simultaneously measured the LPFG-induced discharge time and the transmission spectra both in hot air and AC-20 asphalt binder at five different temperatures, 60, 80, 100, 135, and 170 Celsius. An electromechanical rotational viscometer was also used to measure the viscosities, 0.15–213.80 Pa·s, of the same asphalt binder at the above five temperatures. A non-linear regression analysis was performed to convert LPFG-induced discharge time into viscosities. Comparative analysis shows that the LPFG-induced discharge time agreed well with the viscosities obtained from the rotational viscometer.

## 1. Introduction

The advantages of fiber optic sensors include light weight, small size, immunity to electromagnetic interference (EMI), large bandwidth, environmental ruggedness, and electrical-optical multiplexing. Thus, fiber optic sensors are ideal sensors for the applications of potential smart structures and materials. The fiber optic sensor technology has been applied to the health monitoring of infrastructures [1]. In several industrialized sectors, a viscometer or viscosity sensing system can be an instrument used to measure the viscosity of fluids such as asphalts, motor oils, petroleum products, and solvents. Previous studies of optical viscosity sensors were conducted by using different optical sensing methods. Experimental data published show that the optical viscosity sensing can be based on the use of photodiodes, which is a light-activated switches mechanism [2]. The principle of small angle neutron scattering and dynamic light scattering methods has also been used to measure the viscosity of a colloidal suspension, which consists of a core-shell system made of sterically stabilized silica particles grafted with octadecyl chains in toluene [3]. The sensor concept can be based on the frequency response of a fiber partially submerged in water, sucrose and glycerol solutions of different concentrations, which is sensitive to the viscosity of the above fluids. The viscosity is determined by measuring the vibration of a sinusoidally excited optical fiber probe such as forward light scattering, the bend loss theory, and partially immersed fiber vibrations [4–8]. The optical viscometer has been developed using the laser-induced capillary wave technique to measure the viscosity of distilled water and sulfuric acid with dye of carbon black. This novel micro optical viscosity sensor consists of two deep trenches holding photonic crystal fibers for excitation laser, and two shallow trenches holding the lensed-fibers for probing laser. The optical interference fringe excited by two pulsed laser beams heats the sample surface, and the temporal behavior of surface geometry is detected as a first-order diffracted beam, which contains the information of liquid properties (viscosity and surface tension) [9-11]. The flow of oil films under gravity and centrifugal force may be adapted to give accurate absolute measurements of viscosity for silicon oils [12,13].

In addition, a single optical tweezer can be used as a quantitative tool to perform absolute viscosity of pure water samples on a micrometer-size scale [14]. The wireless magneto-acoustic and magneto-optical sensors have been used to measure the viscosity, temperature and density of water-glycerin mixtures and different grades of motor oil. The sensor oscillations are strongest at the characteristic mechanical resonant frequency of the sensor. Depending upon the physical geometry and surface roughness of the magnetoelastic films, the mechanical sensor-vibrations launch an acoustic wave that can be detected remotely using a hydrophone or microphone. Furthermore, the sensor oscillations act to modulate the intensity of a laser beam reflected from the sensor surface. The sensor vibrations were optically monitored using a photo detector placed in the path of a laser beam back-scattered off the sensor ribbon [15]. The application of molecular rotors, as nonmechanical fluid viscosity sensors, covalently bound to a fiber optic tip technique has been used for optical viscosity sensing particularly in biofluids containing proteins [16]. The above viscosity sensors have been used to measure only low or medium viscous materials, such as distilled water, biofluids, sucrose, glycerol solutions and silicone oils. Their viscosities are less than 160–240 Pa·s at 60 Celsius, which is the viscosity range of an AC-20 asphalt binder. In addition, some viscosity sensors need expensive photonic crystal fibers, special processing, double sensing mechanism—magneto-acoustic and magneto-optical sensors, or complicated mathematical computation such as Fast Fourier Transform.

In this work, we have developed a fiber optical viscometer using an LPFG technique with a capillary tube mechanism. LPFG is extremely sensitive to the refractive index (RI) of the material surrounding the cladding surface, thus allowing it to be used as ambient RI, chemical solution sensors, or chemical concentration indicators. The advantages of LPFGs include their low insertion losses, low back-reflection, polarization independence, and relatively simple fabrication. The other strengths of LPFGs are their simple construction, ease of use, potential capability for on-site, *in vivo*, and remote sensing, easily multiplexed to enable high-throughout screening of chemical reactions, and even disposable and unpackaged sensing. These gratings have offered wide applications in optical communications and sensing systems such as in-fiber band rejection filters and various kinds of sensors for temperature, strain, RI, and other property measurements [17–25]. Base on the combination of the LPFG sensing with a capillary tube mechanism, the LPFG sensor was first immersed in hot air and then in asphalt, and the corresponding discharge time and transmission spectra were measured at the same test temperature. The discharge time was the time spent on 100 mL of the asphalt being measured to flow through the capillary tube when the asphalt reached the immersion level of an LPFG sensor. When the LPFG sensor was immersed in the constant volume (100 mL) AC-20 asphalt binder, a wavelength shift was observed and acquired using LabVIEW software and GPIB controller connected with an optical spectrum analyzer (OSA). As soon as the preheated 100 mL asphalt reaches the mark of an LPFG, the time need was used to determine the discharge time. At the discharge time, the corresponding wavelength shift could be obtained from the spectra. An OSA was used since we would like to acquire and analyze transmission spectra data such as light intensity and wavelength shift. In fact, if the 100 mL discharge time is the only measurand, usually an LPFG with optical power meter or a light reflectivity change from a cutting plane of optical fiber could be used. It only needs an optical fiber (or fiber grating), a light emitting diode and a light detector. Optical power of transmission or reflection light from LPFG or fiber cutting plane changes when the surface of asphalt reaches to the fiber cutting plane. For the first time to our knowledge, the LPFG technique with a capillary tube mechanism was able to successfully yield a comparable viscosity sensing performance.

## 2. Principle of Operation and Fabrication

In general, an LPFG usually is a photo-induced periodic modulation of refractive index along the core of a single-mode fiber, with a typical perturbation of Δn ∼ 10^−4^, periods between 100 μm–1 mm and length of 2–4 cm. The LPFG couples light from a guided fundamental core mode (LP*_01_*) to different forward-propagating cladding modes (HE*_1m_*) in an optical fiber, which is given by phase-matching condition [25]:

(1) $$\beta_{\text{core}}^{01}-\beta_{\text{cladding}}^{1m}=\frac{2\pi }{\Lambda }\;\;\;\;\;m=2,3,4,.\ldots$$

where $\beta_{\text{core}}^{01}$ and $\beta_{\text{cladding}}^{1m}$ are propagating constants of the fundamental core mode and *m*th cladding mode, respectively, and Λ is the period of grating. The coupling of the light into the cladding region generates a series of resonant bands centered at wavelength *λ_m_* in the transmission spectrum, since a cladding mode is rapidly attenuated in the fiber due to scattering losses. The center wavelengths *λ_m_* of an attenuation band are solutions of the following equation [25]:

(2) $$\lambda_{m}=[{\bar{n}}_{\text{core}}^{01}(n_{1},\;n_{2},\;\lambda_{m})-{\vec{n}}_{\text{cladding}}^{1m}(n_{2},\;n_{s},\;\lambda_{m})]\Lambda$$

where ${\bar{n}}_{\text{core}}^{01}(n_{1},\;n_{2},\;\lambda_{m})$ is the effective index of the fundamental core mode at the wavelength of *λ_m_*, which is also dependent on the core refractive index *n*_1_ and cladding refractive index *n*_2_. Also ${\vec{n}}_{\text{cladding}}^{1m}(n_{2},\;n_{s},\;\lambda_{m})$ is the effective refractive of the *m*th cladding mode at the wavelength *λ_m_*, which is related to cladding refractive index *n*_2_ and the refractive index of the surrounding medium *n_s_*. When the concentration or the refractive index of the surrounding medium changes, also ${\vec{n}}_{\text{cladding}}^{1m}(n_{2},\;n_{s},\;\lambda_{m})$ changes and a shift in the central wavelength can be obtained. This unusual feature of LPFG has recently drawn several experimental and theoretical investigations [20,23–28] towards the use of the LPFG as a highly sensitive refractive index sensor or chemical sensor. They have demonstrated that, as the surrounding refractive index *n_s_* is changed from *n_s_* = 1 to *n_s_* = 1.44, the major effect is a blue-shift of the center wavelengths of the attenuation bands, which in particular occurs in the longest wavelengths bands. In this case, by proper selection of grating parameters (period and cladding mode), wavelength shifts as large as 100 nm could be observed and sensitivity can be greatly enhanced. The principle of sensing is based on the total internal reflection experienced by the cladding mode between the interface of the cladding and the surrounding medium. For 1.45 < *n_s_* < 1.46, an abrupt change in the spectrum is occurred in which the cladding is expected to no longer support discrete guided modes, leading to the coupling spectrum spreads (broadband radiation modes) and nearly disappears for the highest order modes. At this point, the wavelength shifts of maximum coupling to shorter wavelengths could be observed and the highest sensitivity is shown by the highest order mode. For case of *n_s_* > 1.46 the cladding mode no longer experiences total internal reflection and the coupling to cladding mode structures reappears. It is shown that for higher order modes, as the index increases, the peak transmission loss increases and the wavelength shifts slightly increase (red-shift) or remain the same.

The cladding modes are very sensitive to change in the refractive index of the ambient (surrounding) environment, particularly when the ambient refractive index higher than that of the cladding index is referred to as a leaky cladding mode [17,25–28]. The leaky cladding modes in LPFG arising from Fresnel reflection have been extensively studied by several authors [17,25–28]. In this situation, either wavelength shifts or amplitude changes of resonance peak wavelengths can be used to sense the external environment change. Previous experiments are normally conducted by dropping a series of ambient refractive-index oils and some mixture of them (RI from 1.00–1.60) upon the LPFG. The wavelength shifts are found to be nearly constant (about 1–2 nanometers) when the ambient refractive index is greater than the RI, about 1.46, of cladding material. As an example, for the highest order of spectrum with an ambient refractive index of 1.64, it is shown that there is +2 nm wavelength shifts and the peak of LPFG spectrum becomes deeper [17]. Furthermore, the air, water, and ethylene glycol or index matching oil have been used as different materials in a series of ambient refractive indexes from 1.00 to 1.72, and the results show that the 1,560 nm resonance wavelength shifts a couple nanometers when the ambient refractive index is between 1.46 and 1.72 [25]. In a similar reported experimental study, several long-period gratings were fabricated in hydrogen-loaded Corning Flexcore fiber and then submerged into various refractive index oils (in the RI range of 1.00 to 1.73), and results illustrate that the 1,520 nm resonance wavelength shifts a couple more nanometers for the ambient refractive indexes of 1.49, 1.57, 1.66, and 1.73 [26]. For the surrounding refractive indices higher than that of the cladding, the resonance wavelength shifts have been shown a considerably reduced sensitivity and shifting about a few nanometers [27]. Those experiments have shown that if the refractive index is much higher than the cladding, such as 1.54, 1.57, 1.66, or 1.73, for the higher order modes the resonance wavelengths of the LPFG are slightly increased (about several nanometers) and the peaks of LPFG attenuation bands become deeper [25–27].

The materials used in most previous studies are not high viscous fluids, such as AC-20 asphalt, and are normally tested at room temperature, which are different from the five test temperatures we used, *i.e.*, 60, 80, 100, 135, and 170 Celsius. In this paper, we focused on the use of LPFGs as level sensors and the wavelength shifts were not used to quantify the viscosity values or refractive indexes of asphalt binders. For our experiment, the temperature effect can be ignored since we measured the wavelengths both in hot air and asphalt at the same temperatures, such as 60, 80, 100, 135, and 170 Celsius. It has been reported that the RI of an AC-20 asphalt could be in the range of 1.64–1.66 (vide infra) which is higher than that of the cladding, but there is no enough information regarding the RI testing conditions and the type of asphalt (bitumen) [29]. With this refractive index, the responses of resonance peaks of the LPFG spectrum to an AC-20 asphalt are expected to become deeper.

**Figure 1.**
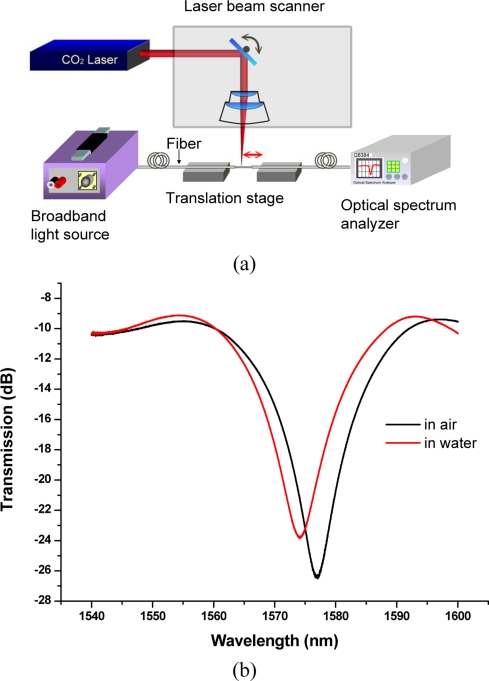
**(a)** Schematic for experimental setup of LPFGs fabrication; **(b)** transmission spectrum of an LPFG sensor in air and immersed in water at 25 Celsius.

The fabrication and techniques of LPFGs have been reported elsewhere [25,30,31]. The experimental apparatus consists of a computer-controlled CO_2_ laser associated with a high-speed optical beam scanner, a translation stage, a broadband amplified spontaneous emission (ASE) fiber source and a high-resolution OSA (ANDO AQ6315A) used to monitor *in-situ* the transmission loss as the grating was written. The CO_2_-induced LPFGs studied in the paper were fabricated in hydrogen-free Corning SMF-28 fibers. The LPFGs were about 22 mm long and their grating periods were about 550 μm, written with a laser power of 0.8 W and a total exposure time of about 2 min. The transmission spectrum was interrogated during the writing and its characteristics such as insertion loss, resonance peak wavelength, and peak depth were analyzed after the grating was written. With suitable fabrication parameters such as laser power, exposure time, grating period, and scan speed, the resulting resonance wavelengths ranging from 1,200 nm to 1,600 nm with a greater than 20 dB peak depth were obtained. The experimental setup for LPFGs fabrication is shown in Figure 1(a). Figure 1(b) shows a transmission spectrum of an LPFG sensor in air and immersed in water at 25 Celsius.

In this study, each transmission spectrum was referenced to the background spectrum of a bare fiber in air. The 3 dB bandwidth was determined by finding the peak of the ASE spectrum, and rising by 3 dB on each side. The spectral width of the ASE spectrum was determined by the separation of these two points because each has a power spectral density equal to one half the peak power spectral densities. The resonance wavelength was calculated as the average of two wavelengths determined in the 3 dB bandwidth measurements.

## 3. Experimental and Modeling

The test material, the sensing mechanism of an LPFG-based viscometer, and the use of non-linear regression model are described in this section.

### 3.1. Material and Viscosity Sensing System

Since asphalt is one of the most popular paving materials, an AC-20 asphalt binder was used as an example of a viscous material. The viscosity range of an AC-20 asphalt binder should be 200 ± 40 Pa·s. The AC-20 asphalt is refined from crude oil and it is thus not a polymer-modified asphalt but rather a petroleum asphalt cement. Since asphalt binders are viscoelastic and temperature-dependent materials and their properties, such as viscosity and refractive index, depend on several factors including the crude oil source, petroleum refining techniques, using additives or not, test temperature, and age effect. Thus, our material, an unmodified asphalt binder refined from crude oil, meeting the requirement of 200 ± 40 Pa·s at 60 Celsius is classified as AC-20 asphalt. The refractive index of the AC-20 asphalt was rarely reported from the current available journals, books, or internet resources. As reported in [29], the refractive index of the asphalt is about 1.64–1.66 and the material described therein could be unmodified asphalts, AC-10 (100 ± 20 Pa·s at 60 Celsius) or AC-20 asphalt binders. However, there is no enough information regarding the RI testing conditions and the type of asphalt (bitumen) [29]. Viscosity can simply be defined as resistance to flow of a fluid. Viscosity grading of asphalt binders usually is based on viscosity measurement at 60 Celsius. To the best of our knowledge, for those samples of asphalt binders we studied in this work, there are no theoretical results of the viscosity being made. They have to be measured using the rotational viscometer, which is the most common viscosity measurement device for asphalt binders or high viscosity materials in the field of civil engineering. The basic equation for absolute viscosity or dynamic viscosity is: $\eta =\frac{\tau }{\frac{d\gamma }{dt}}$, where, τ= shear stress, $\frac{d\gamma }{dt}$ = strain rate. Strain rate is the same as the velocity gradient. In addition, the kinematic viscosity, $\nu =\frac{\eta }{\rho }$, which is the ratio of absolute viscosity to mass density. Thus, kinematic viscosity can be obtained by converting absolute viscosity into kinematic viscosity [32].

**Figure 2.**
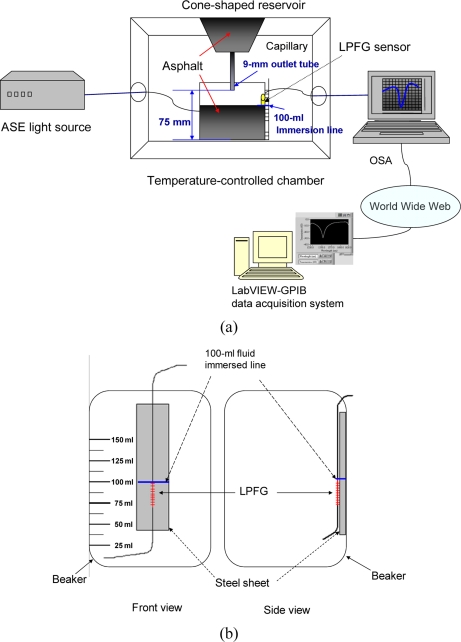
Schematic for **(a)** an LPFG-based viscometer; **(b)** an LPFG bonded on a steel sheet glued on the wall of a beaker.

Figure 2 shows the schematic for the LPFG-based viscometer. This optical viscometer consists of a LPFG sensor, a broadband ASE fiber source, a high-resolution OSA (ANDO AQ6331), a temperature-controlled chamber, a computer with LabVIEW 8.5 software package and GPIB controller (NI-488.2, GPIB-USB-HS), and a cone-shaped reservoir with capillary where gravitational force could cause the liquid, such as asphalts, to flow through the capillary tube [32,33]. The diameter of capillary outlet tube is 9 mm and the distance between the outlet tube and the bottom of the beaker is 75 mm [see Figure 2(a)]. A home-made data acquisition system running with LabVIEW software package and GPIB interface, was used to acquire and analyze the transmission spectrum data of the LPFG from the OSA. During the course of data taking, we kept the eye’s horizon line be matching with the 100-mL level mark and the discharge time, independently measured by using a stopwatch (within 0.1 s error), was the time duration of 100 mL of fluid, such as asphalt, to flow through the capillary tube when the fluid reached the immersion level of an LPFG sensor [see Figure 2(b)]. We marked the immersion level, at the top of each LPFG, to ensure all LPFGs have been exactly 100% immersed in 100 mL asphalt. The immersion level of an LPFG was at the same 100 mL level of a beaker [see Figure 2(b)]. The LPFG sensor was first immersed in hot air and then in asphalt, and its transmission spectra was measured at the same temperature with the OSA. When the LPFG sensor was immersed in the constant volume (100 mL) AC-20 asphalt binder, a wavelength shift was observed and acquired by the data acquisition system. As soon as the asphalt reaching the mark of an LPFG, the time needed between empty and 100 mL was used to determine the discharge time. At the discharge time, the corresponding wavelength shift could be obtained from the spectra. The sensing length of the LPFG fibers used in this work was around 2 cm. Our data acquisition system was designed to detect the wavelength shift of the LPFG at high speed as the test liquid reached the top sensing level of the LPFG within a few mm depths. For precise viscosity measurement, we kept experimental setup in hot air and asphalt measurement at a constant temperature (within 0.1 °C fluctuation). Since we measured the same test temperature for both in hot air and asphalt, the temperature effect causing wavelength change between room temperature and test temperature is the same and can be eliminated.

Furthermore, AC-20 asphalts possess a viscosity of 200 ± 40 Pa·s at 60 Celsius; it is hard to purge the asphalt out of the LPFG after testing. Thus, we used several LPFGs with different resonant wavelengths as disposal sensing components for the optical viscometer. The LPFGs were bonded, with high-temperature-resistance adhesive tape, at both ends on a small straight steel sheet, which was glued to the wall of a beaker to minimize the bending of the fiber. Therefore, we controlled to minimize the variations of test results, not influenced by temperature, strain or bending effects, as much as possible. Such an optical LPFG-based viscometer was capable of measuring simultaneously the discharge time and transmission spectra of a fluid at different temperatures. We measured the discharge time and transmission spectra for the AC-20 asphalt binder using this LPFG-based viscometer at 60, 80, 100, 135, and 170 Celsius. The viscosity of this asphalt was also tested at 60, 80, 100, 135, and 170 Celsius using an electromechanical type of Brookfield rotational viscometer (RV, HB DV-II+ Pro) based on the method of AASHTO T316 [34]. The LPFG-induced discharge time, in seconds, were proposed to be converted into absolute viscosities, which were reference viscosities and measured from RV at the same temperatures.

### 3.2. Non-Linear Regression Model

The relationship between the discharge time, *t*, from the LPFG viscometer and the viscosities, *η*, from the Brookfield rotational viscometer was established using non-linear regression analysis. The Carreau-Yasuda-like model has been successfully used in the rheology area (such as polymers, asphalts), and thus was selected for the use of a non-linear regression model [35,36]. The Carreau-Yasuda-like model was as follows:

(3) $$\eta (t)=\eta_{\infty }+(\eta_{0}-\eta_{\infty })\times {\left[1+{\left(\lambda t\right)}^{\alpha }\right]}^{\frac{\beta -1}{\alpha }}$$

This model has five adjustable parameters, *α*, *β*, *λ*, *η_o_*, and *η_∞_*. We fitted this model to the data, viscosity (*η*) obtained from rotational viscometer, as a function of LPFG-based discharge time (*t*) and calculated the above parameters. For non-linear regression modeling, the spreadsheet-like software, Excel Solver was performed. The tangent mode set for estimate, the forward step used for derivates and the Newton method selected as search technique were carried out.

## 4. Results and Discussion

### 4.1. Viscosity Measurements

The LPFG-based viscometry for asphalt binders was conducted with three samples. The average discharge time (see Table 1) from the LPFG viscometer were 7,934, 1,448, 446, 35, 24 seconds at 60, 80, 100, 135, and 170 Celsius, respectively.

**Figure 3.**
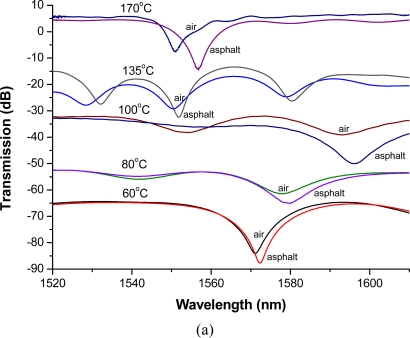

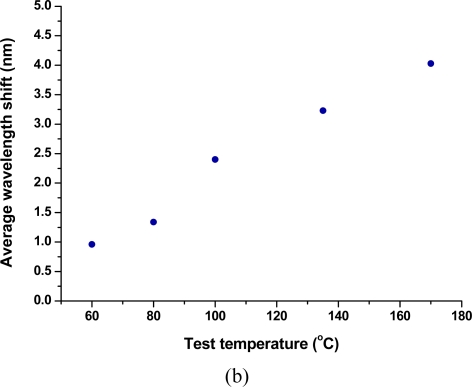
**(a)** Transmission spectra and **(b)** average wavelength shift of the LPFG sensors in hot air and asphalt at several temperatures.

This viscometer combines an LPFG level sensor and a capillary tube mechanism. The 100 mL discharge time is the key mersurand. The LPFGs were used as a level sensor with an OSA since we could acquire and analyze transmission spectra data such as light intensity and wavelength shift. However, the wavelength shifts of LPFGs varied probably due to the fabrication parameter factors and without quality-control process. The transmission spectra and average resonance wavelength shifts of the LPFG sensors in hot air and asphalt at several temperatures are plotted in Figure 3. Obviously, when the LPFGs were immersed in asphalt, the resonance wavelengths of the sensors were shifted to the longer wavelength. Figure 3(a) shows a plot of transmission spectra of the LPFG sensors in hot air and asphalt with increasing changes in temperature (from bottom to top). The transmission spectra have been offset for a better presentation. Based on the results of Table 1, there is no clear relationship between wavelength shifts and viscosities at different temperatures. We did not try to prepare the LPFGs having similar responses to the refractive index change because the information of wavelength shifts were not used to obtain the viscosity of the samples used in the experiment. It can be seen that the average wavelength shifts increased as the test temperatures increased. The asphalt has been reported having a refractive index, about 1.64, higher than the cladding [29]. Figure 3(b) displays the average wavelength shifts, with a 3 dB bandwidth method, about 0.96, 1.34, 2.40, 3.23, and 4.03 nm at 60, 80, 100, 135, and 170 Celsius, respectively. The amount of resonance wavelength shifts of AC-20 asphalt binder, under the condition of the five temperatures, were found to be about 1–4 nm and showed the trend of slightly increase with increasing temperature, indicating that the corresponding refractive indices were more than 1.46 since the wavelength shifts were measured as positive values. It is noted that the amount of wavelength shifts (∼1–4nm) found here were also matched with some previously reported results for the case of the RIs greater than 1.46 or more [25–27].

**Figure 4.**
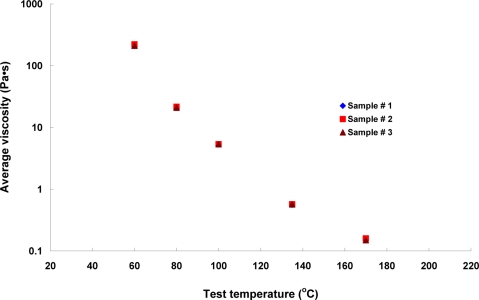
Viscosities of asphalt samples from a Brookfield rotational viscometer at 60, 80, 100, 135, and 170 Celsius.

Thus, our experimental results presented here reveal that the refractive index of AC-20 asphalt could be more than 1.64 when it was tested more than 60 Celsius of temperature and this value was temperature-dependent. Furthermore, Table 1 also shows the viscosity results obtained from the Brookfield RV. The viscosity values for three samples, and each sample with five replicates at 60, 80, 100, 135, and 170 Celsius were plotted in Figure 4.

The average viscosity values at 60, 80, 100, 135, and 170 Celsius were 213.800, 21.127, 5.350, 0.567, and 0.153 Pa·s, respectively. The quantitative comparison of discharge time from LPFG-based viscometer and viscosities from RV was based on the analysis of root mean square (RMS). The range of RMS values for LPFG-based and rotational viscometers were 0.29–27.33 s and 0.05–53.96 Pa·s, respectively. The coefficient of variation, normalization in statistics, indicates the variation of data from the discharge time of the LPFG-based viscometer and viscosities from RV were 0.05–2.72% and 0.19–3.77%, respectively. Thus, the proposed method—LPFG-based viscometer possessed the potential to measure the viscosity of a fluid.

### 4.2. Comparative Analysis

**Figure 5.**
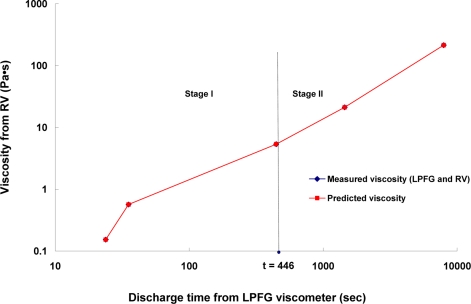
Comparative plot of LPFG-RV-measured viscosity and predicted viscosity.

Based on the results from the optical and electromechanical viscometers, the log-log-scale relationship between the viscosity (*η*) of this asphalt binder using RV versus the discharge time (*t*) from LPFG viscometer is shown in Figure 5 (see diamond icon). The non-linear regression was performed in two stages, since when the discharge time was about greater than 446 seconds (corresponding 100 Celsius, *t* = 446 s), the asphalt binder behaved or flowed like a Newtonian fluid. The viscosity grading of asphalt binders, $\eta =\frac{\tau }{\frac{d\gamma }{dt}}$ is almost a constant. Thus, the slope of viscosity-discharge time line is close to a constant and this could be seen in Figure 5. The error sum of squares (SSE) of the non-linear regression models with stages I and II were 9.366E-07 and 6.368E-10, respectively.

Figure 5 shows that the comparative plot of LPFG-RV-measured viscosities (see diamond icon) agreed well with the predicted viscosities (see rectangular icon). The following equations show the two-stage results for non-linear regression analysis using the Carreau-Yasuda-like model:

(4) $$\eta (t)=(-91.0159)+(-32.3291+91.0159)\times {\left[1+{\left(0.010157\;t\right)}^{0.656007}\right]}^{\frac{0.943056-1}{0.656007}};\;\text{if}\;t\;<\;446\;s\;\;(\text{Stage}\;\text{I})$$

(5) $$\eta (t)=(-1.63459)\times {\left[1+{\left(0.002646\;t\right)}^{0.882737}\right]}^{\frac{-0.727609-1}{0.882737}};\;\;\text{if}\;t\;\geq \;446\;s\;\;(\text{Stage}\;\text{II})$$

## 5. Conclusions

Studies presented in this paper successfully illustrate the feasibility of fabricating a class of fiber optical viscometer based on the LPFG sensor written by CO_2_ laser pulses. The realization of the sensor is through the measurement of discharge time for 100 mL AC-20 asphalt binder to flow through the capillary tube whereas the asphalt reached the LPFG sensor and induced a wavelength shift. Data were collected and analyzed at five different temperatures (60, 80, 100, 135, and 170 Celsius). For the first time we report the data of viscosity of AC-20 asphalt binder at five different temperature and present the explicit formula between the viscosity and discharge time for this sample. Experimentally, the LPFG-induced discharge time was converted into viscosities using the Carreau-Yasuda-like model and results were compared with those measured from RV at the same temperatures. Theoretically, the two-stage non-linear regression analysis was applied to compare the LPFG-RV-measured viscosities with the predicted ones. Our results show that the computational predictions agreed well with the experimental data. Although in this paper the LPFG was used just as a level sensor, it can be turned to a simple way of measuring the discharge time that finally has a link to the viscosity of the test liquid. Thus, in this study we have successfully demonstrated a novel LPFG capillary tube viscometer and its feasibility of measuring the viscosity of AC-20 asphalt binder at five different temperatures which has yet to be reported.

The proposed LPFG-based viscometer could potentially perform two simultaneous physical measurands, the viscosity and refractive index, of a fluid. To our knowledge, this is the first time an LPFG sensor associated with a capillary tube mechanism has successfully shown a comparable viscosity sensing performance. Such a highly sensitive fiber-optic viscosity sensor is suitable for use in various fields of applications, such as civil, food, chemical and biological, mechanical, petroleum, and aerospace engineering. The advantage of this type of sensor is relatively simple of construction, compact, low cost, and ease of use. Moreover, the sensor has the potential capability for on-site, *in vivo*, and remote sensing, and has the potential use for disposable sensors.

## Figures and Tables

**Table 1.** Experimental data of LPFG-based and rotational viscometers.

	**Method**	**Test temperature, °C**				

		**60**	**80**	**100**	**135**	**170**
**LPFG viscometer**	100 mL Discharge time, s	7,930.0	1,407.9	445.1	34.5	23.4
		7,935.0	1,465.4	446.1	34.6	24.1
		7,938.0	1,466.3	445.5	36.2	23.9

	Mean, s	7,934.3	1,447.6	445.5	35.2	23.8

	RMS^1^, s	3.30	27.33	0.41	0.78	0.29

	CV^2^, %	0.05	2.31	0.11	2.72	1.51

**Rotational viscometer**	Viscosity, Pa·s^3^	209.40	20.80	5.34	0.56	0.15
		221.40	21.60	5.36	0.57	0.16
		210.60	20.98	5.35	0.57	0.15

	Mean, Pa·s	213.80	21.13	5.35	0.57	0.15

	RMS, Pa·s	53.96	3.43	0.08	0.05	0.05

	CV, %	3.09	1.99	0.19	1.02	3.77

1 RMS = root mean square.

2 CV = coefficient of variation.

3 Pa·s = 10 poises = 1,000 centipoises
